# The effectiveness and outcomes of epidural analgesia in patients undergoing open liver resection: a propensity score matching analysis

**DOI:** 10.1186/s12871-024-02697-1

**Published:** 2024-09-02

**Authors:** Isarapong Pianngarn, Worakitti Lapisatepun, Maytinee Kulpanun, Anon Chotirosniramit, Sunhawit Junrungsee, Warangkana Lapisatepun

**Affiliations:** 1 Department of Anesthesiology, Faculty of Medicine, Chiang Mai University, 110 Inthawarorot Road, T. Sriphum, A. Muang, Chiang Mai, 50200 Thailand; 2 Department of Surgery, Division of Hepatobilliary Pancreatic Surgery, Faculty of Medicine, Chiang Mai University, 110 Inthawarorot Road, T. Sriphum, A. Muang, Chiang Mai, 50200 Thailand; 3 Clinical Surgical Research Center, Chiang Mai University, 110 Inthawarorot Road, T. Sriphum, A. Muang, Chiang Mai, 50200 Thailand

**Keywords:** Epidural analgesia, Open liver resection, Effectiveness and safety, Opioid consumption, Propensity score matching

## Abstract

**Background:**

Open liver resection necessitates a substantial upper abdominal inverted-L incision, resulting in severe pain and compromising patient recovery. Despite the efficacy of epidural analgesia in providing adequate postoperative analgesia, the potential epidural-related adverse effects should be carefully considered. This study aims to compare the efficacy and safety of continuous epidural analgesia and intravenous analgesia in open liver resection.

**Methods:**

A retrospective study was conducted, collecting data from patients who underwent open liver resection between 2007 and 2017. Propensity score matching was implemented to mitigate confounding variables, with patients being matched in a 1:1 ratio based on propensity scores. The primary outcome was the comparison of postoperative morphine consumption at 24, 48, and 72 hours between the two groups. Secondary outcomes included pain scores, postoperative outcomes, and epidural-related adverse effects.

**Results:**

A total of 612 patients were included, and after matching, there were 204 patients in each group. Opioid consumption at 24, 48, and 72 hours postoperatively was statistically lower in the epidural analgesia group compared to the intravenous analgesia group (*p* < 0.001). However, there was no significant difference in pain scores (*p* = 0.422). Additionally, perioperative hypotension requiring treatment, as well as nausea and vomiting, were significantly higher in the epidural analgesia group compared to the intravenous analgesia group (*p* < 0.001).

**Conclusions:**

Epidural analgesia is superior to intravenous morphine in terms of reducing postoperative opioid consumption within the initial 72 h after open liver resection. Nevertheless, perioperative hypotension, which necessitates management, should be approached with consideration and vigilance.

**Trial registration:**

The study was registered in the Clinical Trials Registry at www.clinicaltrials.gov/, NCT number: NCT06301932.

## Background

Liver resection is the surgical procedure for treating benign and malignant liver tumors, primarily performed through an open approach with a right inverted L-shaped incision or right subcostal incision. Consequently, the upper abdominal incision is associated with severe postoperative pain and delayed patient recovery [1]. Thoracic epidural analgesia (TEA) is a conventional technique commonly used to control postoperative pain after major abdominal surgery, including open liver resection [2–5]. This technique provides adequate postoperative pain control, reduces narcotic use without increasing the length of stay or perioperative complications, and decreases postoperative morbidities and other serious complications [4, 6–9].

However, many recent studies have focused on the safety of neuraxial anesthesia and the deleterious epidural-related adverse effects in patients undergoing liver resection due to the potential risk for postoperative coagulopathy and the likelihood of developing serious complications, including epidural hematoma [10, 11]. Furthermore, perioperative hypotension is also a common epidural catheter-related adverse effect that may require treatment, either through a significantly greater volume of intravenous fluid administration or the use of vasoactive drugs. These concerning adverse effects might be the causes of delayed removal of the epidural catheter and delayed patient ambulation during the postoperative period, which is the key to successful and enhanced recovery after liver resection [12].

This study aims to compare the efficacy of continuous thoracic epidural analgesia and intravenous morphine administration and evaluate the safety of continuous thoracic epidural analgesia in patients who underwent open liver resection.

## Methods

### Study population

**Fig. 1:**
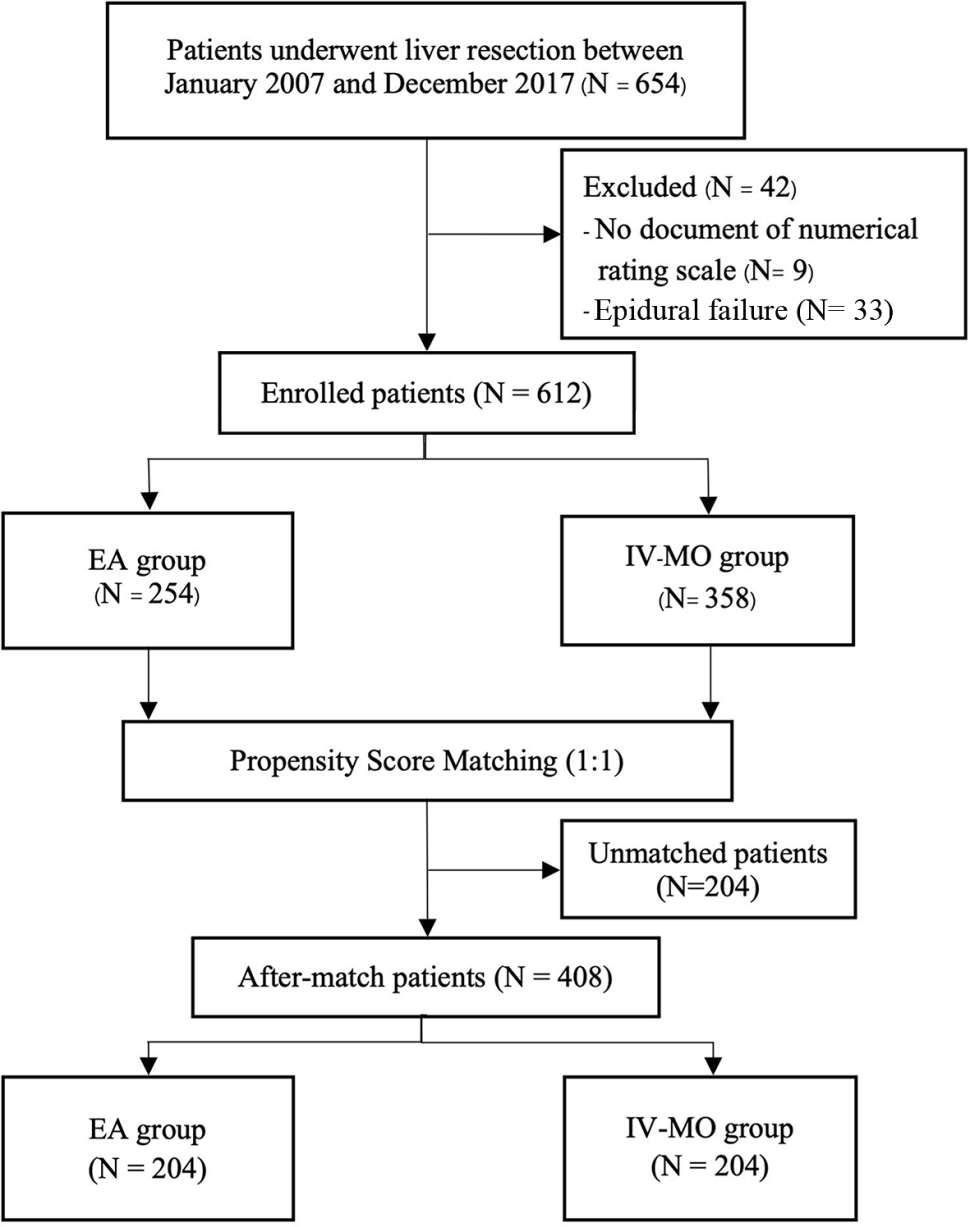
Flowchart of 612 patients undergoing open liver resection

This study was conducted as a retrospective observational cohort study by reviewing and collecting the database of all patients aged over 18 who underwent an elective open liver resection in a tertiary hospital center. The data were extracted from the electronic medical record system between January 2007 and December 2017. The study was conducted according to the guidelines of the Declaration of Helsinki and ethically approved by the Institutional Review Board of the Faculty of Medicine, Chiang Mai University (IRB number: ANE-2562-06771, approved on December 23, 2019). Patient-informed consent was waived by the Ethics Committee due to the retrospective nature of the study, and the analysis used anonymous clinical data. A total of 654 patients were initially enrolled in this study; however, those with a failure of epidural analgesia (*n* = 33) and those without documentation of numerical rating scores (*n* = 9) were excluded, resulting in a final participant of 612 patients, as shown in Fig. 1.

In terms of operational definitions, open major liver resection was characterized by the removal of four or more liver segments, whereas a minor liver resection was defined as the removal of no more than four segments. These definitions adhere to the anatomical classification outlined in the Brisbane 2000 Terminology of Liver Anatomy and Resections [13]. The failure of epidural analgesia was defined as inadequate analgesia, such that the analgesic level could not be tested or there was no sensory block after adequate dosing of local anesthesia following the initial placement, resulting from catheter dislodgement or any reason for early discontinuation of the epidural catheter [14].

### Data collection and outcome measurements

For each patient, demographic data, including age, body mass index (BMI), gender, American Society of Anesthesiologists (ASA) classification, and preoperative laboratory investigations, were collected. Intraoperative data, including type of liver resection, intravenous fluid administration, the incidence of hypotension, the use of vasopressors or inotropic drugs, operating time, and estimated blood loss, were also assessed. Postoperative data regarding postoperative opioid consumption, the numerical rating scale (NRS), and postoperative outcomes, including surgical and non-surgical complications and epidural-related complications, were also reported.

All eligible patients were categorized into two groups: the continuous thoracic epidural analgesia group (EA group) and the intravenous morphine group (IV-MO group). Additionally, the EA group was divided into epidural analgesia with local anesthetics with opioids (EA-O group) and epidural analgesia with local anesthetics without opioids (EA-L group). The primary outcome was to compare postoperative morphine consumption at 24, 48, and 72 hours. Moreover, the numerical rating scale at 24, 48, and 72 hours and postoperative outcomes, such as length of hospital and ICU stays, the need for ventilator support, and epidural-related complications, were also reported as secondary outcomes. Postoperative pain was assessed using the numerical rating scale, in which patients reported pain on a scale from 0 to 10 at 24, 48, and 72 hours postoperatively.

In our institute, all liver resections were performed by the hepatobiliary surgeons utilizing general anesthesia, with or without continuous epidural analgesia. Most open liver resections were performed using a standard inverted-L incision. Continuous thoracic epidural analgesia was administered to patients undergoing liver resection based on the anesthesiologist’s decision and the patient’s conditions. Patients with pre-operative coagulopathy and thrombocytopenia were considered relative contraindications for implementing epidural analgesia. Experienced anesthesiologists or senior residents performed continuous thoracic epidural analgesia under supervision at mid-thoracic level (T8-T9 or T9-T10) in awake patients. The loss of resistance technique or epidural wave form was utilized to confirm the correction of the epidural placement. Continuous thoracic epidural analgesia was not routinely used during the intraoperative period, depending on the anesthesiologist’s discretion.

Continuous epidural analgesia was usually employed after liver resection in patients without contraindications for epidural catheter insertion, with 0.1-0.125% bupivacaine with or without the addition of opioid (fentanyl 1 µg/mL) at a continuous infusion rate ranging from 5 to 12 mL/hour; however, early interruption or delayed usage of the epidural catheter was necessary in some patients who developed postoperative hypotension. When the patients experienced postoperative severe pain (NRS ≥ 7), rescue analgesia comprising 3–4 mg of intravenous morphine or 25–50 µg of intravenous fentanyl was administered to those in the EA-L group and IV-MO group. Additionally, for the patients in the EA-O group, intravenous tramadol at 50 mg was administered as rescue postoperative pain relief. The calculation of morphine milligram equivalents was used to convert the dosage of other intravenous opioids into intravenous milligram morphine equivalents [15].

### Statistical analysis

For the continuous variables, the differences between the two groups were compared by using the Student *t*-test for normal distribution data and the Mann-Whitney U test for non-normal distribution data. Continuous data with a normal distribution were reported as the mean (± standard deviation), while non-normally distributed variables were reported as the median (interquartile range). Categorical variables were analyzed by Fisher’s exact test, which is presented as numbers with percentages.

Propensity score matching was applied to adjust confounding factors by indication and contraindication and reported according to Lonjon et al. [16] The propensity scores were calculated using a multivariable logistic regression model. The epidural analgesia group (EA group) and intravenous morphine group (IV-MO group) were matched by propensity scores that were generated for each case based on the following baseline covariates: age, co-morbidity, ASA classification, the extent of the resected liver, and pre-operative white blood cell count (WBC), prothrombin time (PT), international normalized ratio (INR), albumin, and total bilirubin (TB). These variables were used to attain the similar baseline characteristics that occurred before performing epidural analgesia and had the potential to influence either the patient’s status or the attending anesthesiologist’s decision to utilize epidural analgesia. The patients with missing data in matching variables at a rate of 4.4% and those who could not be matched were excluded from the analysis. A 1:1 nearest-neighbor match with a standard caliper width of 0.2 was performed to generate a matched cohort. The analysis of the absolute standardized difference was used to evaluate the balance after propensity score matching of all pre-operative covariates between the EA group and the IV-MO group.

Postoperative morphine consumption and numerical rating scale at 24, 48, and 72 hours between the EA group and the IV-MO group were compared by using the Mann-Whitney U test and repeated measures ANOVA, respectively. Moreover, subgroup analysis was performed by using ANOVA with Bonferroni’s test that compared postoperative opioid consumption in the EA-L, EA-O, and IV-MO groups. A *P*-value < 0.05 was considered statistically significant. The data were analyzed using STATA version 16.0 (StataCorp LP, College Station, Texas, USA).

## Results

A total of 654 patients undergoing liver resection met the inclusion criteria. Forty-two patients were excluded from this study due to no documentation of NRS (*n* = 9), and failure of epidural analgesia was noted (*n* = 33), leaving 612 patients. Among these 612 patients, 254 received epidural analgesia (EA group) and 358 received intravenous morphine (IV-MO group) for postoperative pain control. After matching, 204 patients in the EA group were matched with 204 patients in the IV-MO group (Fig. 1).

### Baseline variables and outcomes before matching

Epidural analgesia was utilized in 254 patients (41.5%) in the study population. The median age of the study population was 56 years old (IQR 48–63), and 56.8% of the population were males. The body mass index was similar among the groups. The majority of the indication for liver resection is primary liver tumors (75.1%), including hepatocellular carcinoma and cholangiocarcinoma, and accordingly, approximately 55.4% of the study population underwent a major liver resection. Patient characteristics were significantly different between the two groups in the type of liver resection, preoperative white blood cell count, prothrombin time, international normalized ratio, albumin, and total bilirubin. Epidural analgesia was performed more frequently in major liver resection than in minor liver resection (171 (67.3%) versus 83 (32.7%), *P < 0.001).* Baseline characteristics of all patients undergoing liver resection prior to match are reported in Table 1.

**Table 1:** Baseline characteristics before and after propensity score matching in patients undergoing open liver resection

Characteristics	Before PS matching		*P*	SDD	After PS matching		*P*	SDD
	EA group (*N* = 254)	IV-MO group (*N* = 358)			EA group (*N* = 204)	IV-MO group (*N* = 204)		
Age (year)	55.8 (±11.5)	55.4 (±12.2)	0.637	-0.039	55.9 (± 11.4)	55.4 (± 12.0)	0.666	-0.043
Male	151 (59.5)	198 (55.3)	0.321	0.084	123 (60.3)	106 (52.0)	0.110	-0.068
BMI (kg/m^2^)	22.3 (±3.4)	22.6 (±3.7)	0.522	0.053	22.4 (±3.6)	22.6 (±3.5)	0.585	0.054
ASA classification			0.955	0.020			0.817	0.039
I II III	33 (16.2) 155 (76.0) 16 (7.8)	44 (12.3) 152 (74.5) 17 (8.3)			36 (17.7) 151 (74.0) 17 (8.3)	32 (15.7) 156 (76.5) 15 (7.8)		
Co-morbidity	123 (48.4)	198 (55.3)	0.101	0.138	106 (52.0)	106 (52.0)	1.000	0.001
Hepatitis profiles			0.861	-0.043			0.185	-0.017
Hepatitis B Hepatitis C Hepatitis B & C	31 (12.2) 6 (2.4) 4 (1.6)	44 (12.3) 5 (1.4) 5 (1.4)			23 (11.7) 5 (2.5) 3 (1.5)	31 (15.3) 1 (0.5) 1 (0.5)		
Preoperative diagnosis			0.173	0.010			0.198	-0.028
Primary liver malignant Liver metastasis Benign tumor	182 (71.9) 19 (7.5) 52 (20.6)	279 (77.9) 17 (4.8) 62 (17.3)			143 (70.4) 18 (8.9) 42 (20.7)	157 (77.0) 10 (4.9) 37 (18.1)		
Child-Pugh classification			0.230	0.144			0.856	0.074
A B C	214 (84.6) 33 (13.0) 6 (2.4)	283 (79.5) 58 (16.3) 15 (4.2)			171 (84.2) 26 (12.8) 6 (3.0)	166 (82.2) 30 (14.6) 6 (3.0)		
Type of liver resection			< 0.001	-0.426			0.547	-0.070
Minor liver resection Major liver resection	83 (32.7) 171 (67.3)	191 (53.4) 167 (46.6)			81 (39.7) 123 (60.3)	88 (43.1) 116 (58.9)		
Hilar resection	68 (26.8)	77 (21.5)	0.148	-0.123	52 (25.5)	52 (25.5)	1.000	0.011
Vascular reconstruction	31 (12.3)	30 (8.4)	0.132	-0.127	21 (10.3)	23 (11.3)	0.873	-0.001
Preoperative testing								
Hemoglobin (g/dL) WBC (10^3^/µL) PT (second)	12.4 (± 1.8) 7.1 (5.8–8.8) 11.4 (± 1.1)	12.3 (± 1.3) 7.7 (6.4 − 9.6) 11.5 (± 1.2)	0.369 0.004 < 0.001	-0.074 0.253 0.368	12.3 (± 1.8) 7.3 (5.8–9.3) 11.4 (± 1.1)	12.5 (± 1.8) 7.4 (6.4–8.8) 11.4 (± 1.2)	0.160 0.996 0.576	0.014 -0.062 0.056
PTT (second)	31.2 (± 3.6)	31.8 (± 6.7)	0.153	0.123	31.2 (± 3.8)	31.2 (± 6.7)	0.346	0.094
INR	1.1 (± 0.1)	1.1 (± 0.2)	0.880	0.378	1.1 (±0.11)	1.1 (±0.17)	0.879	0.015
Platelet count (10^3^/µL) Albumin (g/dL) Globulin (g/dL)	277.2 (± 102.2) 3.8 (± 0.5) 3.6 (± 0.7)	266.0 (± 119.3) 3.6 (± 0.7) 3.8 (± 0.9)	0.799 < 0.001 0.098	0.021 -0.415 0.140	277.2 (± 102.2) 3.8 (± 0.6) 3.7 (± 0.8)	266.0 (± 119.3) 3.8 (± 0.6) 3.6 (± 0.8)	0.309 0.737 0.609	-0.009 -0.033 -0.051
AST (U/L) ALT (U/L) TB (mg/dL) DB (mg/dL) ALP (U/L) BUN (mg/dL) Creatinine (mg/dL)	38.0 (26.0–63.5) 31.0 (20.0–55.0) 0.7 (0.5–1.2) 0.2 (0.1–0.6) 109.0 (77.0–186.0) 12.0 (9.0–14.0) 0.9 (0.70–1.0)	39.0 (28.0–68.0) 32.5 (22.0–59.0) 0.8 (0.5–1.5) 0.2 (0.1–0.7) 125.0 (85.0–210.0) 11.0 (9.0–14.0) 0.9 (0.7–1.1)	0.167 0.146 0.005 0.736 0.088 0.259 0.078	0.087 0.067 0.159 0.099 0.199 -0.020 0.210	38.5 (26.0–64.0) 32.5 (20.0–56.0) 0.7 (0.5–1.3) 0.2 (0.1–0.6) 108.5 (80.0–195.0) 12.0 (9.0–14.0) 0.87 (0.70–1.00)	37.0 (27.0–60.0) 30.0 (21.0–54.0) 0.7 (0.5–1.3) 0.2 (0.1–0.6) 116.0 (79.0–199.0) 11.0 (8.0–14.0) 0.80 (0.70–1.00)	0.674 0.936 0.440 0.735 0.432 0.083 0.178	-0.072 -0.053 -0.002 -0.022 0.075 -0.033 0.050

Values are presented as number (%), mean (± standard deviation), and median (interquartile range)

*Abbreviations* EA group: Epidural analgesia group; IV-MO group: Intravenous Morphine group; PS: Propensity Score; SDD: Standardized Difference; BMI: Body Mass Index; ASA classification: American Society of Anesthesiology classification; WBC: White Blood Cell Count; PT: Prothrombin Time; PTT: Partial Thromboplastin Time; INR: International Normalized Ratio; AST: Aspartate aminotransferase; ALT: Alanine aminotransferase; TB: Total Bilirubin; DB: Direct Bilirubin; ALP: Alkaline Phosphatase; BUN: Blood Urea Nitrogen

### Baseline variables and outcomes after matching

**Fig. 2:**
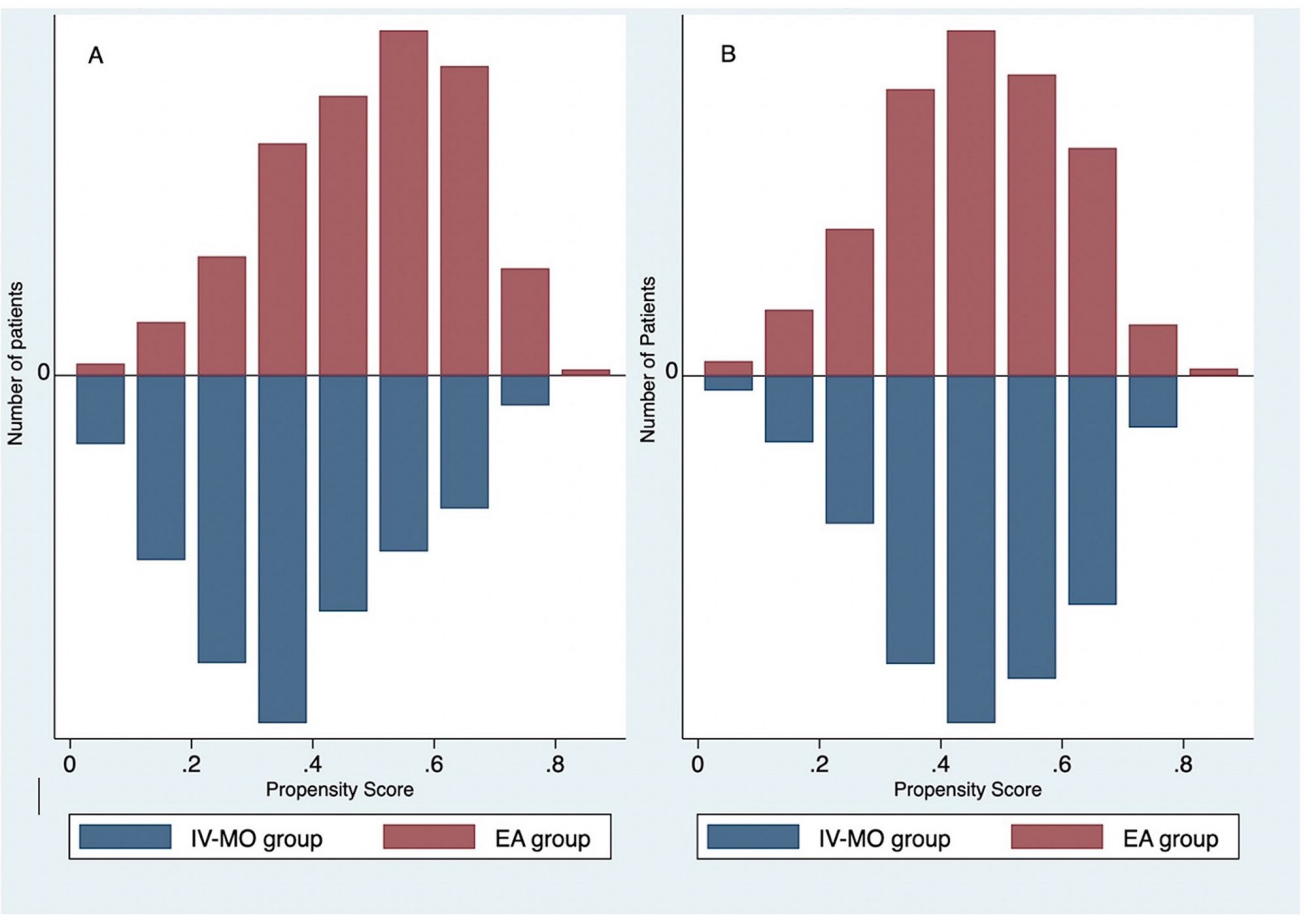
Distribution of propensity scores before matching (**A**) and after matching (**B**)

**Fig. 3:**
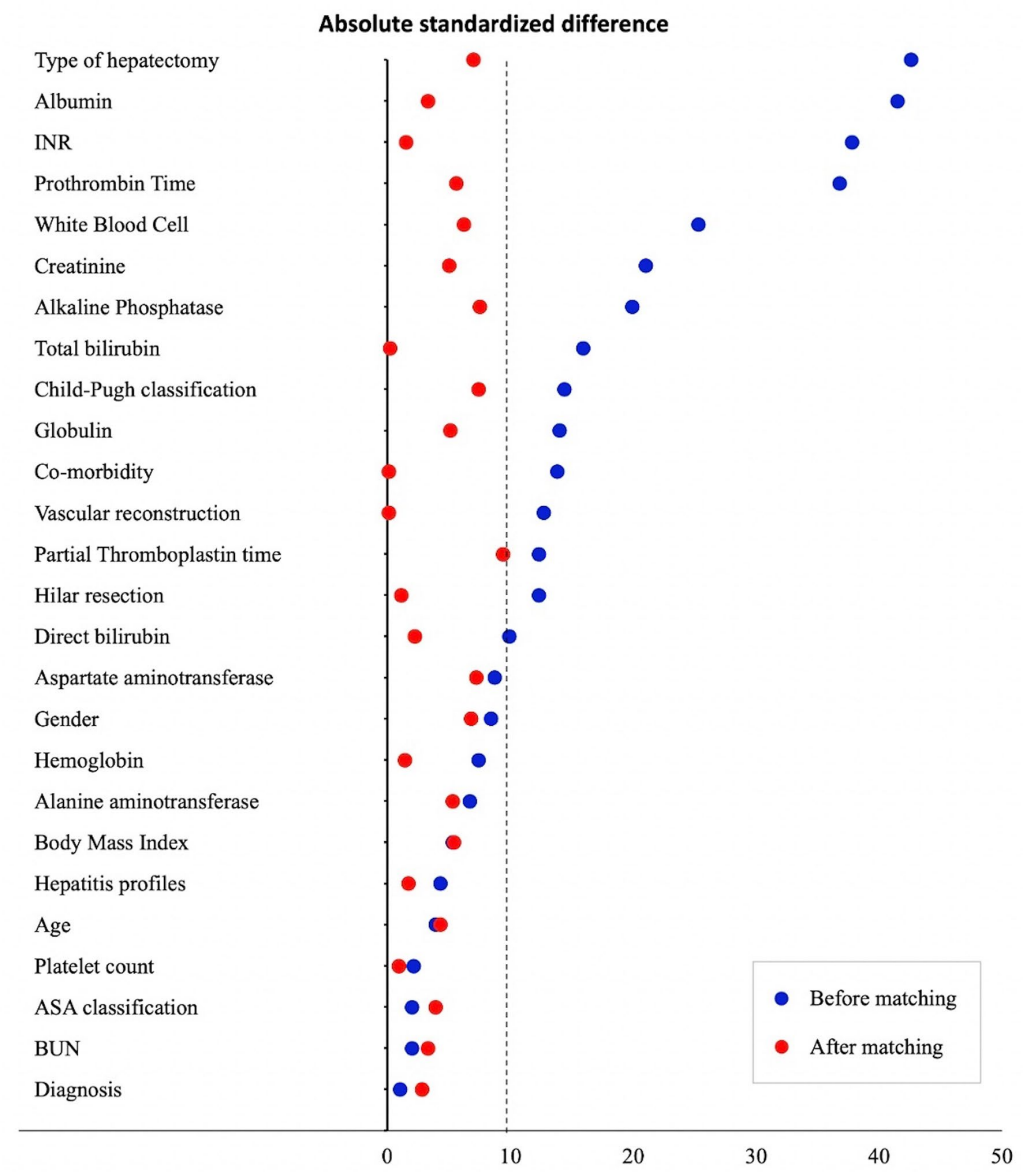
Absolute standardized difference before and after propensity score matching for baseline covariates comparing between epidural group and intravenous morphine group

After matching, 204 patients were in each group, which was similarly balanced on baseline characteristics between the two groups, as shown in Table 1. The propensity scores were calculated with mulvariable logistic analysis by using baseline covariats that showed mean propensity scores in each group were nearly equally (0.46 ± 0.15 vs. 0.45 ± 0.15, *P = 0.800).* Moreover, the distribution of propensity scores across the two groups before and after matching was acceptable, as shown in Fig. 2. The graphical representation of the absolute standardized difference across covariates prior to and after propensity score matching. All covariates after matching were less than 10% of the standardized threshold, which represented an adequate balance of baseline covariates between the EA group and the IV-MO group (Fig. 3).

**Fig. 4:**
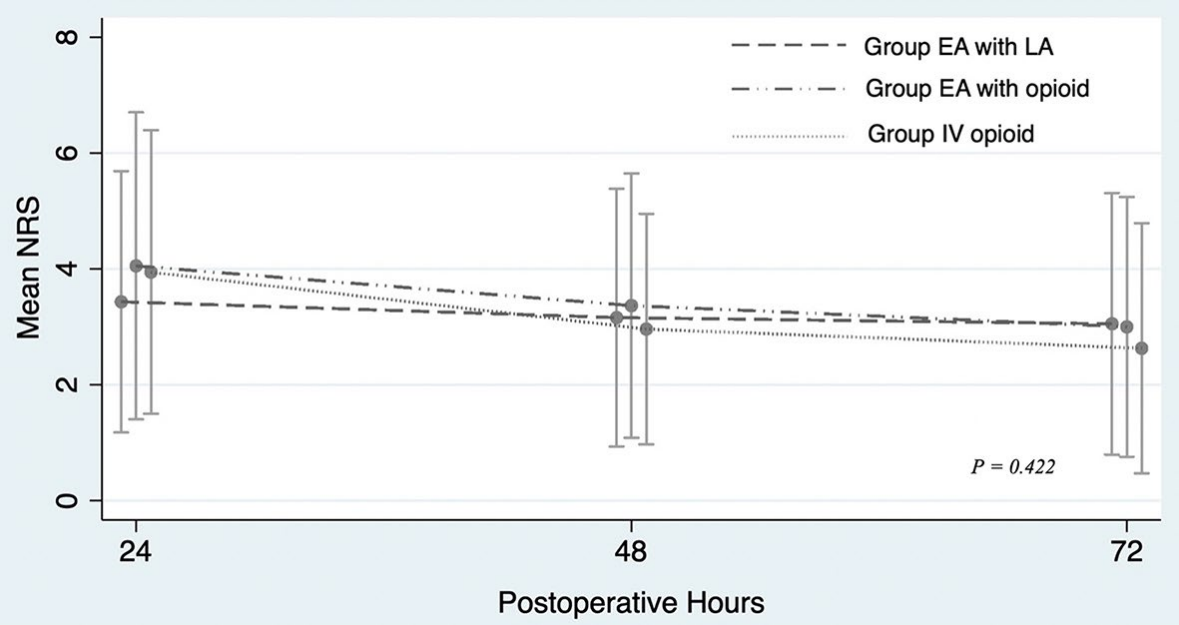
Postoperative numerical rating scale in patients undergoing open liver resection

The EA group exhibited significantly lower morphine consumption compared to the IV-MO group at 24, 48, and 72 hours postoperatively (*P < 0.001*, Table 2). The EA group was further divided into two subgroups, which were EA with opioids (EA-O) and without opioids (EA-L). The morphine consumption in both subgroups was significantly lower than that of the IV-MO group, although no significant difference was observed between EA-O and EA-L (Table 3). The mean numerical rating scale of the three groups was assessed at 24, 48, and 72 hours postoperatively and compared by using repeated measure ANOVA, revealing no statistically significant difference (*P = 0.422*), as illustrated in Fig. 4.

**Table 2:** Postoperative intravenous opioid consumption in patients undergoing open liver resection (*N* = 408)

Opioid consumption (Morphine equivalent)	EA group (*N* = 204)	IV-MO group (*N* = 204)	*P*	Median difference (95% CI)
Opioid consumption (mg/day)				
POD 1 (0–24 hours) POD 2 (24–48 hours) POD 3 (48–72 hours) Total 3 Days (0–72 hours)	5.0 (0.0–12.0) 0.0 (0.0–5.0) 0.0 (0.0–5.0) 10.0 (3.0–21.0)	16.0 (10.0–24.0) 10.0 (3.0–20.0) 3.0 (0.0–10.0) 35.0 (21.0–48.0)	< 0.001 < 0.001 < 0.001 < 0.001	-11.0 (-13.2, -8.8) -10.0 (-11.9, -8.1) -3.0 (-4.7, -1.3) -25.0 (-29.4, -20.6)

Values are presented as median difference

*Abbreviations* EA group: Epidural analgesia group; IV-MO group: Intravenous Morphine group; POD: Postoperative day; 95% CI: 95% Confidence Interval

**Table 3:** Subgroup analysis of postoperative intravenous opioid consumption in patients undergoing open liver resection (*N* = 408)

Opioid consumption	EA-L (*N* = 84) vs. EA-O (*N* = 117) group		EA-O (*N* = 117) vs. IV-MO (*N* = 207) group		EA-L (*N* = 84) vs. IV-MO (*N* = 207) group	
	Mean difference (95%CI^*^)	P*	Mean difference (95%CI^*^)	P*	Mean difference (95%CI^*^)	P*
POD 1 (0–24 hours) POD 2 (24–48 hours) POD 3 (48–72 hours) Total 3 Days (0–72 hours)	-1.0 (-2.9, 1.0) -1.0 (-0.5, 2.5) -1.0 (-1.7, 1.3) -0.2 (-4.2, 3.8)	0.958 0.093 0.201 0.265	-9.6 (-11.7 -7.4) -8.1 (-10.0, -6.3) -2.6 (-4.2, -1.1) -20.3 (-24.6, -16.0)	< 0.001 < 0.001 0.005 < 0.001	-10.6 (-13.1, -8.1) -7.1 (-9.3, -4.9) -2.9 (-4.6, -1.2) -20.6 (-25.7, -15.5)	< 0.001 < 0.001 < 0.001 < 0.001

Values are presented as mean difference

*Adjustment for multiple comparison with Bonferroni’s correction

*Abbreviations* EA-L group: Epidural with local anesthetic without opioid group; EA-O group; Epidural with local anesthetic with opioid group; IV-MO group: Intravenous Morphine group; POD: Postoperative day; 95% CI: 95% Confidence Interval; VS: Versus

The incidence of intraoperative hypotension was significantly higher in the EA group (73.0% vs. 55.1%, *P* < *0.001*), which was associated with significantly higher usage of vasopressors and inotropic drugs (67.2% vs. 52.0%, *P* < 0.001). However, the amounts of intravascular volume administration and estimated blood loss were not different between the two groups (2525.0 mL vs. 2500.0 mL, *P* = 0.657, and 640.0 mL vs. 700.0 mL, *P* = 0.818, respectively) (Table 4).

**Table 4:** Intraoperative data in patients undergoing open liver resection (*N* = 408)

Intraoperative variables	Post-matching (*n* = 408)		*P*
	EA group (*n* = 204)	IV-MO group (*n* = 204)	
Intravenous fluid administration (mL) Crystalloid Colloid	2525.0 (1850.0–3400.0) 1725.0 (1250.0-2475.0) 600.0 (100.0-1175.0)	2500.0 (1760.0–3225.0) 1700.0 (1200.0-2500.0) 515.0 (200.0-1200.0)	0.657 0.723 0.615
Hypotension	146 (73.0)	109 (55.1)	< 0.001
Vasopressors & inotropic drugs	137 (67.2)	106 (52.0)	< 0.001
Urine output (mL)	370.0 (210.0-650.0)	352.5 (200.0-540.0)	0.333
Estimated blood loss (mL)	640.0 (400.0–1200.0)	700 (400.0–1100.0)	0.818
Intraoperative transfusion	73 (35.8)	89 (44.1)	0.105
Operating time (minute)	332.2 (± 9.7)	326.7 (± 9.0)	0.676
Extubation	142 (71.4)	137 (68.4)	0.662

Values are presented as number (%), mean (± standard deviation), and median (interquartile range)

*Abbreviations* EA group: Epidural group; IV-MO group: Intravenous Morphine group

The length of hospital stays, incidence of ICU admission, and the length of ICU stays were also not statistically different (*P  = 0.500*,* 0.424*,* and 0.479* respectively) (Table 5). Surgically related complications were not significantly different between both groups. However, the number of non-surgical-related complications was statistically higher in the EA group compared to the IV-MO group, including postoperative hypotension (20.1% vs. 1.5%; *P* < 0.001), and postoperative nausea and vomiting (4.9% vs. 0%; *P* < 0.001). In addition, one patient who received epidural analgesia with opioids developed respiratory depression. Lastly, no epidural hematoma was reported in our study.

**Table 5:** Postoperative outcomes in patients undergoing open liver resection (*N* = 408)

Postoperative outcomes	EA group (*N* = 204)	IV-MO group (*N* = 204)	*P*
Length of hospital stay (day)	8.0 (7.0–12.0)	8.0 (7.0–12.0)	0.500
Length of ICU stay (day)	0.0 (0.0–2.0)	1.0 (0.0–2.0)	0.424
ICU admission	89 (45.2)	96 (49.2)	0.479
Surgical complications			
Reoperation Intraabdominal hemorrhage Surgical site infection	2 (1.0) 2 (1.0) 10 (5.1)	4 (2.1) 2 (1.0) 7 (3.6)	0.685 1.000 0.621
Non-surgical complications			
Hypotension required treatment Nausea and vomiting required treatment Postoperative coagulopathy Atelectasis Pneumonia Pleural effusion Pulmonary embolism Liver failure Acute kidney injury Urinary tract infection Urinary retention Sepsis	41 (20.1) 10 (4.9) 55 (28.7) 5 (2.5) 5 (2.5) 6 (3.1) 2 (1.0) 6 (3.1) 14 (7.1) 0 (0.0) 5 (2.5) 6 (3.0)	3 (1.5) 2 (0.9) 46 (23.6) 2 (1.0) 4 (2.0) 8 (4.1) 0 (0.0) 3 (1.5) 10 (5.1) 2 (1.0) 4 (2.0) 6 (3.0)	< 0.001 < 0.001 0.298 0.449 1.000 0.787 0.499 0.503 0.528 0.499 1.000 1.000

Values are presented as number (%), and median (interquartile range)

*Abbreviations* EA group: Epidural analgesia group; IV-MO group: Intravenous Morphine group; ICU: Intensive Care Unit

## Discussion

This study constitutes one of the most extensive single-center investigations on the efficacy and outcomes of epidural analgesia for open liver resection. Thoracic epidural analgesia was employed for postoperative pain management in 41.5% of the patients with a low failure rate (5%) and no serious catheter-related complications. Our results reveal that epidural analgesia significantly reduces morphine consumption within the initial 72 hours postoperatively compared to intravenous opioid administration. These findings are consistent with prior research [2, 4, 5, 17, 18]. A systematic review and meta-analysis have shown that thoracic epidural analgesia provides superior pain control for patients undergoing open liver resection, compared to patient-controlled analgesia 48 hours after surgery [17]. Nonetheless, we found that no significant differences were observed in pain scores on the numerical rating scale between the EA group and IV-MO group, which contrasts with findings from previous studies [3–5]. This result could be caused by the retrospective nature of our study, potentially affecting the accuracy of pain score measurements, whether before or after morphine administration, and for both resting and movement-induced pain. Additionally, the pain score is inherently subjective and can vary widely among patients.

In our center, the utilization rate of epidural analgesia in open liver resection was relatively high (41.5%) compared with other studies, where usage ranged from 5.9 to 13.9% [19, 20]. The observed failure rate of epidural analgesia in our study was 5.0%, markedly lower than the approximately 20-30% reported in other research [21, 22]. This difference may be due to our center’s high rate of epidural analgesia utilization and the procedures being performed or supervised by experienced anesthesiologists.

The majority of participants in our study were diagnosed with primary liver tumors and had pre-operative coagulopathy, hypoalbuminemia, and hyperbilirubinemia. These factors may introduce potential selection biases regarding the application of epidural analgesia. Additionally, the clinical decision to use TEA was based on the patient’s preoperative condition and the extent of liver resection, resulting in significant differences in baseline characteristics between the groups. These disparities make it difficult to compare and interpret the outcomes of the EA group and the IV-MO group. To mitigate these biases, propensity score matching was employed to achieve good comparability between the groups with well-balanced baseline characteristics. Our study differs from prior retrospective studies, often characterized by smaller sample sizes and unadjusted selection bias [23, 24], by having a large sample size from a single center and a high utilization rate of epidural analgesia.

Although epidural analgesia is beneficial for various abdominal surgeries, particularly upper abdominal surgery, and is commonly performed in open liver resection [4, 25], there are special concerns regarding the safety of epidural analgesia in liver resection due to epidural-related complications, such as epidural hematoma, spinal cord injury resulting in permanent paraplegia, epidural abscess, localized pain at the epidural site, and intrathecal catheterization [26, 27]. Spinal cord injury can occur, particularly in patients who are extremely age, obese, or have diabetes [28]. Therefore, it is crucial to carefully weigh the risks and benefits when considering epidural analgesia in these patients.

Our study found no significant difference in postoperative complications between groups, similar to other reports [3, 29]. However, the incidence of hypotension appeared to be higher in the EA group than in the IV-MO group, consistent with a prior study that reported 75% of patients with epidural analgesia developed perioperative hypotension [24] and required greater amounts of intravenous fluid until 72 hours postoperatively [30]. Moreover, the combination of epidural analgesia with general anesthesia is often used during the intraoperative period, which increases the risk of intraoperative hypotension and the need for vasopressors and inotropic drugs, similar to previous studies [21, 24]. The possible causes of intraoperative hypotension are peripheral vasodilatation due to sympathetic nervous system blockade and maintaining a low central venous pressure technique during the parenchymal transection phase to reduce blood loss [24, 31].

Perioperative hypotension in patients receiving epidural analgesia is a common adverse event related to regional anesthesia and is associated with a higher rate of postoperative nausea and vomiting in the EA group in our study. Various mechanisms have been proposed to explain postoperative nausea and vomiting in patients receiving regional anesthesia, including perioperative hypotension precipitating brain stem and gut hypoperfusion, resulting in the release of emetogenic substances, as well as the use of epidural opioids and high levels of anesthetic blockage [32].

Moreover, acute kidney injury (AKI) after liver resection is caused by maintaining low central venous pressure and the utilization of epidural analgesia, leading to hypotension and reduced renal perfusion pressure [33]. A large retrospective study reported an incidence of acute kidney injury occurring 8.2–12.0% that was associated with epidural analgesia, age ≥ 60 years old, chronic renal failure, major liver resection, and blood transfusion requirement [33, 34]. Nevertheless, our result showed there was no significant difference in AKI incidence between the groups, consistent with previous studies that found no significant difference in renal failure and postoperative creatinine levels between patients with and without epidural analgesia [35]. This is likely due to the routine administration of adequate intravascular fluid resuscitation following parenchymal transection, guided by either central venous pressure or stroke volume variation monitoring.

Postoperative coagulation disturbance caused by transient liver dysfunction after liver resection should be considered due to potential bleeding complications, including catheter-related epidural hematoma, which is the most serious epidural-related complication [36, 37]. Although the incidence of epidural hematoma is extremely rare (1:150,000) [26, 31], spinal hematomas after epidural analgesia in cirrhotic patients have been reported [38]. Moreover, patients with pre-operative inadequate hemostasis, pre-existing liver cirrhosis, extensive liver resection, small remnant of liver volume, and significant blood loss greater than 1000 ml during surgery have increased potential risks of postoperative coagulopathy, leading to delayed removal of the epidural catheter after liver resection [10, 25, 37, 39, 40]. These factors can exacerbate postoperative coagulopathy, leading to an increased risk of bleeding and complications. In our study, precautions were taken to mitigate these risks, including careful intraoperative management of blood loss, regular monitoring of coagulation parameters, and timely administration of blood products as needed. Despite the high utilization rate of epidural analgesia in our study population, none developed epidural hematomas.

The length of hospital stays was not significantly different between the two groups, aligning with previous studies [3, 4, 17, 36] due to the fact that the day of epidural catheter removal in our study was postoperative day 3 in patients without postoperative coagulopathy, in accordance with the Enhanced Recovery After Surgery (ERAS) pathway for liver resection [6]. In contrast, some studies [19, 41] reported longer hospital stays for patients receiving epidural analgesia, which was associated with delayed epidural catheter removal and is a controversial issue in the ERAS protocol for liver resection [12]. A recent randomized controlled trial by John et al. found shorter postoperative hospital stays in patients receiving intravenous patient-controlled analgesia (IV-PCA) compared to those with epidural analgesia. The authors argued that IV-PCA is non-inferior to epidural analgesia in terms of pain relief, simplicity of use, cost-effectiveness, ease of implementation, and reduced time consumption compared to epidural analgesia [41].

Continuous thoracic epidural analgesia is an effective technique for providing excellent postoperative pain control in patients undergoing liver resection, as recommended in the PROSPECT group [42]. However, perioperative hypotension is a drawback of this technique, which could potentially delay ambulation. Accordingly, optimizing fluid administration, ensuring adequate postoperative pain control, and employing multimodal analgesia are crucial to preventing hypotension and facilitating enahanced recovery after surgery [12, 43].

Due to the nature of a retrospective study, an important factor in this study is the typically unavoidable biases in the study. Although we utilized propensity score matching to adjust the selection bias, residual confounding factors related to indications and contraindications remained. Additionally, our institute lacked standard protocols for prescriping of local anesthetics in epidural analgesia, and we did not control the rate and concentration of local anesthetics. Lastly, the routine application of thoracic epidural analgesia in liver resection is not standard practice in some institutions due to concerns regarding the high rate of epidural failure, postoperative coagulation disturbance, and other adverse effects; accordingly, generalizations of our study should be considered.

## Conclusions

To conclude, thoracic epidural analgesia is superior to intravenous opioid administration in reducing postoperative opioid consumption within the first 72 hours postoperatively. While perioperative hypotension requiring treatment is a consideration, the incidence of epidural-related hypotension during the postoperative period can be minimized with adequate intravascular volume replacement after liver parenchymal resection and an understanding of the physiological changes after liver surgery. Our study suggests that continuous thoracic epidural analgesia can be effectively and safely performed in patients undergoing open liver resection without contraindications. Further high-quality randomized controlled trials could be conducted to determine the effectiveness and safety of epidural analgesia in liver resection.

## Data Availability

The datasets supporting the conclusion of this study are available from the corresponding author upon request.

## Abbreviations

TEA: Thoracic Epidural Analgesia
IRB: Institute Review Board
BMI: Body Mass Index
ASA: American Society of Anesthesiologists (ASA) classification
NRS: Numerical Rating Scale
EA group: Epidural Analgesia group
IV-MO group: Intravenous Morphine group
EA-O group: Epidural Analgesia with Local Anesthetics with Opioids group
EA-L group: Epidural Analgesia with Local Anesthetics without Opioids group
ICU: Intensive Care Unit
IQR: Interquartile Range
AKI: Acute Kidney Injury
ERAS: Enhanced Recovery After Surgery
IV-PCA: Intravenous Patient-Controlled Analgesia
